# Serum Neutralizing Activity of mRNA-1273 against SARS-CoV-2 Variants

**DOI:** 10.1128/JVI.01313-21

**Published:** 2021-11-09

**Authors:** Angela Choi, Matthew Koch, Kai Wu, Groves Dixon, Judy Oestreicher, Holly Legault, Guillaume B. E. Stewart-Jones, Tonya Colpitts, Rolando Pajon, Hamilton Bennett, Andrea Carfi, Darin K. Edwards

**Affiliations:** a Moderna, Inc., Cambridge, Massachusetts, USA; Loyola University Chicago

**Keywords:** COVID-19, SARS-CoV-2 variants of concern, mRNA-1273, neutralization

## Abstract

The emergence of severe acute respiratory syndrome coronavirus 2 (SARS-CoV-2) variants has led to growing concerns over increased transmissibility and the ability of some variants to partially escape immunity. Sera from participants immunized on a prime-boost schedule with the mRNA-1273 COVID-19 vaccine were tested for neutralizing activity against several SARS-CoV-2 variants, including variants of concern (VOCs) and variants of interest (VOIs), compared to neutralization of the wild-type SARS-CoV-2 virus (designated D614G). Results showed minimal, statistically nonsignificant effects on neutralization titers against the B.1.1.7 (Alpha) variant (1.2-fold reduction compared with D614G); other VOCs, such as B.1.351 (Beta, including B.1.351-v1, B.1.351-v2, and B.1.351-v3), P.1 (Gamma), and B.1.617.2 (Delta), showed significantly decreased neutralization titers ranging from 2.1-fold to 8.4-fold reductions compared with D614G, although all remained susceptible to mRNA-1273-elicited serum neutralization.

**IMPORTANCE** In light of multiple variants of SARS-CoV-2 that have been documented globally during the COVID-19 pandemic, it remains important to continually assess the ability of currently available vaccines to confer protection against newly emerging variants. Data presented herein indicate that immunization with the mRNA-1273 COVID-19 vaccine produces neutralizing antibodies against key emerging variants tested, including variants of concern and variants of interest. While the serum neutralization elicited by mRNA-1273 against most variants tested was reduced compared with that against the wild-type virus, the level of neutralization is still expected to be protective. Such data are crucial to inform ongoing and future vaccination strategies to combat COVID-19.

## INTRODUCTION

As the coronavirus disease 2019 (COVID-19) pandemic continues to escalate in various parts of the world, several severe acute respiratory syndrome coronavirus 2 (SARS-CoV-2) variants of interest (VOIs) and variants of concern (VOCs) have emerged, including in the United States (B.1.526, Iota; B.1.427/B.1.429), United Kingdom (B.1.1.7, Alpha), Brazil (P.1, Gamma), India (B.1.617.1, Kappa; B.1.617.2, Delta), South Africa (B.1.351, Beta), Uganda (A.23.1), Nigeria (B.1.525, Eta), Peru (C.37, Lambda), Colombia (B.1.621, Mu), and Angola (A.VOI.V2) (1). There is growing concern over these variants based on increased transmissibility and the ability of some variants to partially escape both natural and vaccine-induced immunity. Notably, the B.1.617.2 lineage has been classified as a VOC by the World Health Organization due to evidence of an increased rate of transmission, reduced effectiveness of monoclonal antibody treatment, and reduced susceptibility to neutralizing antibodies (1).

We previously reported that mRNA-1273, a lipid nanoparticle-encapsulated mRNA-based vaccine encoding the spike glycoprotein of the SARS-CoV-2 Wuhan-Hu-1 isolate, induced high neutralizing-antibody titers in phase 1 trial participants (2) and was highly effective in preventing symptomatic and severe COVID-19 (3, 4). Some VOCs or VOIs, including B.1.351 and P.1, reduced neutralizing-antibody levels in a pseudovirus-based model (5). Importantly, however, all variants remained susceptible to mRNA-1273 vaccine-elicited serum neutralization (5). Here, we provide an update on the neutralization activity of vaccine sera against several newly emerged variants, including the Delta variant, B.1.617.2.

## RESULTS

**FIG 1:**
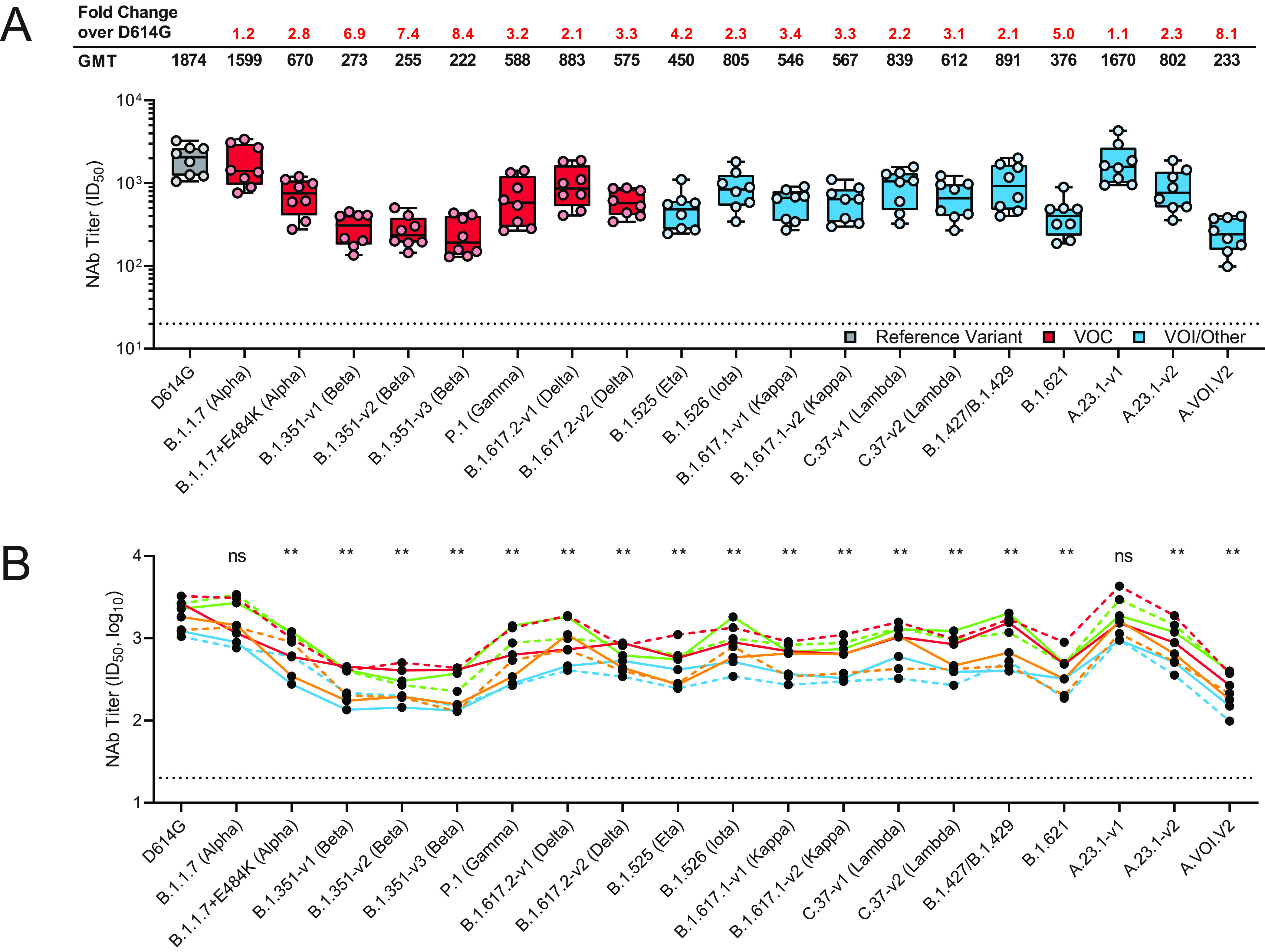
Neutralization of SARS-CoV-2 pseudoviruses in serum samples. Serum samples were obtained from participants in the mRNA-1273 vaccine phase 1 trial on day 36 (7 days after dose 2). A recombinant vesicular stomatitis virus–based pseudovirus neutralization assay was used to measure neutralization. The pseudoviruses tested incorporated D614G or the spike substitutions present in B.1.1.7 (Alpha), B.1.1.7+E484K (Alpha), B.1.351-v1 (Beta), B.1.351-v2 (Beta), B.1.351-v3 (Beta), P.1 (Gamma), B.1.617.2-v1 (Delta), B.1.617.2-v2 (Delta), B.1.525 (Eta), B.1.526 (Iota), B.1.617.1-v1 (Kappa), B.1.617.1-v2 (Kappa), C.37-v1 (Lambda), C.37-v2 (Lambda), B.1.427/B.1.429, B.1.621 (Mu), A.23.1-v1, A.23.1-v2, and A.VOI.V2 (Table 1). The reciprocal neutralizing titers on the pseudovirus neutralization assay at a 50% inhibitory dilution (ID_50_) are shown. In panel A, boxes and horizontal bars denote the interquartile range and the geometric mean titer (GMT), respectively. Whisker end points are equal to the maximum and minimum values below or above the median at 1.5 times the interquartile range (IQR). The GMT fold change over D614G for each variant is shown. In panel B, the colored lines connect the D614G and variant neutralization titers in matched samples. A two-tailed Wilcoxon matched-pairs signed-rank test was performed (**, *P* < 0.01). In both panels, the dots represent results from individual serum samples, and the dotted line represents the lower limit of quantification for titers at 20 ID_50_. Data for B.1.1.7 (Alpha), B.1.1.7+E484K (Alpha), P.1 (Gamma), and B.1.427/B.1.429 were published previously (5). NAb, neutralizing antibody.

We assessed neutralization activity of sera against D614G pseudovirus (predominant variant in 2020), B.1.1.7, B.1.1.7+E484K, B.1.351-v1, B.1.351-v2, B.1.351-v3, P.1, B.1.617.2-v1, B.1.617.2-v2, B.1.525, B.1.526, B.1.617.1-v1, B.1.617.1-v2, C.37-v1, C.37-v2, B.1.427/B.1.429, B.1.621, A.23.1-v1, A.23.1-v2, and A.VOI.V2 (Table 1). Sera from the phase 1 mRNA-1273 clinical trial (8 participants, 1 week following dose 2) were evaluated against each variant (2). Results showed minimal, statistically nonsignificant effects on neutralization titers against B.1.1.7 and A.23.1-v1 compared to D614G (*P = *0.64 and 0.46, respectively) (Fig. 1). In contrast, all other variants examined showed significantly decreased neutralization titers compared with D614G (*P < *0.01) (Fig. 1), although all remained susceptible to mRNA-1273-elicited serum neutralization. Reductions in neutralization titers for these variants ranged from a factor of 2.1 to 8.4 compared with that for D614G (Fig. 1A). Across the 3 versions of the B.1.351 variant tested, 6.9-fold to 8.4-fold reductions in neutralization were observed compared with that for D614G (Fig. 1A). Among all variants tested, the greatest effect on neutralization was observed for A.VOI.V2 and B.1.351-v3 (8.1-fold and 8.4-fold reductions compared with activity against D614G, respectively). More modest 2.1- to 3.4-fold reductions were measured for P.1, B.1.617.2-v1, B.1.617.2-v2, B.1.526, B.1.617.1-v1, B.1.617.1-v2, C.37-v1, C.37-v2, and A.23.1-v2. Intermediate 4.2- and 5.0-fold reductions were seen for B.1.525 and B.1.621, respectively. mRNA-1273-elicited neutralization titers against B.1.1.7, B.1.1.7+E484K, B.1.427/B.1.429, P.1, and B.1.351-v1 observed herein corroborated previous findings (5).

**TABLE 1:** Spike mutations in SARS-CoV-2 variants evaluated in this study

Variant name	WHO nomenclature	Location variant first identified	Amino acid change(s) in spike
D614G		Predominant global variant	D614G
B.1.1.7	Alpha	United Kingdom	ΔH69, ΔV70, ΔY144, N501Y, A570D, D614G, P681H, T716I, S982A, D1118H
B.1.1.7+E484K	Alpha	United Kingdom	ΔH69, ΔV70, ΔY144, E484K, N501Y, A570D, D614G, P681H, T716I, S982A, D1118H
B.1.351-v1	Beta	South Africa	L18F, D80A, D215G, ΔL242, ΔA243, ΔL244, R246I, K417N, E484K, N501Y, D614G, A701V
B.1.351-v2	Beta	South Africa	L18F, D80A, D215G, ΔL242, ΔA243, ΔL244, K417N, E484K, N501Y, D614G, A701V
B.1.351-v3	Beta	South Africa	D80A, D215G, ΔL242, ΔA243, ΔL244, K417N, E484K, N501Y, D614G, A701V
P.1	Gamma	Brazil	L18F, T20N, P26S, D138Y, R190S, K417T, E484K, N501Y, D614G, H655Y, T1027I, V1176F
B.1.617.2-v1	Delta	India	T19R, G142D, E156G, ΔF157, ΔR158, L452R, T478K, D614G, P681R, D950N
B.1.617.2-v2	Delta	India	T19R, T95I, G142D, E156G, ΔF157, ΔR158, L452R, T478K, D614G, P681R, D950N
B.1.525	Eta	Nigeria	Q52R, A67V, ΔH69, ΔV70, ΔY144, E484K, D614G, Q677H, F888L
B.1.526	Iota	United States	L5F, T95I, D253G, E484K, D614G, A701V
B.1.617.1-v1	Kappa	India	T95I, G142D, E154K, L452R, E484Q, D614G, P681R, Q1071H
B.1.617.1-v2	Kappa	India	G142D, E154K, L452R, E484Q, D614G, P681R, Q1071H, H1101D
C.37-v1	Lambda	Peru	G75V, T76I, Δ246-252, D253N, L452Q, F490S, D614G, T859N
C.37-v2	Lambda	Peru	T63I, Δ64-76, Δ246-252, D253N, L452Q, E471Q, F490S, D614G, T859N
B.1.427/B.1.429		United States	S13I, W152C, L452R, D614G
B.1.621	Mu	Colombia	T95I, Y144T, Y145S, ins146N, R346K, E484K, N501Y, D614G, P681H, D950N
A.23.1-v1		Uganda	F157L, V367F, Q613H, P681R
A.23.1-v2		Uganda	R102I, F157L, V367F, E484K, Q613H, P681R
A.VOI.V2		Angola	D80Y, ΔY144, ΔI210, D215G, ΔR246, ΔS247, ΔY248, L249M, W258L, R346K, T478R, E484K, H655Y, P681H, Q957H

## DISCUSSION

Among VOCs tested, serum-elicited neutralization of the B.1.1.7 (Alpha) variant was comparable to that of D614G; a range of significantly reduced neutralization titers compared to D614G were observed for other VOCs, including the B.1.351 (Beta), P.1 (Gamma), and B.1.617.2 (Delta) variants, with reductions ranging from 2.1-fold to 8.4-fold. Results presented here are generally consistent with previous studies examining neutralization activity of mRNA-1273–induced immune sera against VOIs/VOCs (reviewed in reference 6), with similar overall trends using both live-virus and pseudovirus neutralization assays (6, 7). Similar trends in neutralizing activity of VOIs/VOCs by sera from individuals immunized with BNT162b2 were also observed (6, 8–10). A limitation of this study is that differential variant spike incorporation into the various pseudoviruses might impact neutralization results. Nevertheless, these data emphasize the need to continually assess the ability of mRNA-1273 to confer protection against prevalent and emergent VOIs/VOCs. Such preclinical analyses in conjunction with epidemiological monitoring of the incidence and spread of VOCs directly inform strategies around vaccines targeting SARS-CoV-2 variants. As new variants emerge, including those that lead to greater vaccine breakthrough cases, similar analyses could be designed to test vaccine-induced immunity against variants in either animal or clinical studies. Such data are crucial to inform necessary modifications to COVID-19 mRNA vaccines going forward, which may help to mitigate the ongoing spread of SARS-CoV-2 and the emergence of new variants.

## MATERIALS AND METHODS

### Clinical trial.

Healthy adult participants (*n* = 8; age [mean ± standard deviation], 34.8 ± 9.7 years; male, 37.5%) were immunized with mRNA-1273 (100 μg) on a prime-boost schedule, and serum was collected 7 days after the booster (day 36). Study protocols and results have been reported previously (2).

### Recombinant VSV-based pseudovirus assay.

Codon-optimized full-length spike (S) protein of the original Wuhan-Hu-1 isolate with D614G mutation (D614G) was cloned into a pCAGGS vector. This codon-optimized D614G vector was used as a template for site-directed mutagenesis to incorporate the S variants, listed in Table 1. To make SARS-CoV-2 full-length S-pseudotyped recombinant vesicular stomatitis virus ΔG (VSVΔG)-firefly luciferase virus, BHK-21/WI-2 cells (Kerafast) were transfected with the S expression plasmid and subsequently infected with VSVΔG-firefly luciferase as previously described (11). For the neutralization assay, serially diluted serum samples were mixed with pseudovirus and incubated at 37°C for 45 min. The virus-serum mix was subsequently used to infect A549-hACE2-TMPRSS2 cells (12) for 18 h at 37°C before addition of ONE-Glo reagent (Promega) for measurement of the luciferase signal by relative luminescence units (RLUs). The percentage of neutralization was calculated based on the RLUs of the virus-only control and subsequently analyzed using the four-parameter logistic curve in Prism v.8 (GraphPad Software, Inc.).

### Statistical analysis.

A two-sided Wilcoxon matched-pairs signed-rank test was used to compare the same patients against different viruses. Statistical analyses were performed (Prism v.8). Geometric mean titers, lower limit of quantification, and fold change relative to D614G were included.
